# Midwives’ perceptions towards the ministry of health guidelines for the provision of immediate postpartum care in rural health facilities in Uganda

**DOI:** 10.1186/s12884-023-05585-7

**Published:** 2023-04-18

**Authors:** Mariam Namutebi, Gorrette K. Nalwadda, Simon Kasasa, Patience A. Muwanguzi, Dan K. Kaye

**Affiliations:** 1 Department of Nursing, College of Health Sciences, School of Health Sciences, Makerere University, Kampala, Uganda; 2 Department of Epidemiology and biostatistics, College of Health Sciences, School of Public Health, Makerere University, Kampala, Uganda; 3 Department of Obstetrics and Gynecology, College of Health Sciences, School of Medicine, Makerere University, Kampala, Uganda

**Keywords:** Postpartum care, Midwives, Guidelines, Midwives’ perceptions

## Abstract

**Background:**

Guidelines for clinical practice have been part of the Ministry of health’s efforts to improve the quality of care for over two decades. Their benefits have been documented in Uganda. However, having practice guidelines may not always result in their use in care provision. We explored the midwives’ perceptions towards the ministry of health guidelines for providing immediate postpartum care.

**Methods:**

An exploratory descriptive qualitative study was conducted in three districts in Uganda from September 2020 to January 2021. In-depth interviews with 50 midwives from 35 health centers and 2 hospitals in Mpigi, Butambala, and Gomba districts were done. Thematic analysis of data was done.

**Results:**

Three themes emerged; awareness and use of the guidelines, perceived drivers, and perceived barriers to the provision of immediate postpartum care. The subthemes for theme I included; awareness of the guidelines, variations in the postpartum care practices, variations in preparedness to manage women with complications, and varied access to continuing midwifery education. Fear of complications and litigation were the perceived drivers of guideline use. On the other hand, lack of knowledge, busy maternity units, organization of the care, and the midwives’ perceptions about their clients were the barriers to guideline use. Midwives felt that new guidelines and policies regarding immediate postpartum care should be disseminated widely.

**Conclusion:**

The midwives felt that the guidelines were good for the prevention of postpartum complications but their knowledge of the guidelines for the provision of immediate postpartum care was suboptimal. They desired on-job training and mentorship to help them bridge the knowledge gaps. Variations in patient assessment, monitoring, and pre-discharge care were acknowledged and said to be due to a poor reading culture and health facility factors like patient-midwife ratios, unit setup, and prioritization of labor.

## Introduction

Immediate postpartum care (from the delivery of the baby to 24 h after delivery) is part of the facility based continuum of perinatal care for women and newborns [1]. It entails the monitoring of vital signs, assessment of the maternal and newborn wellbeing, health education for the mother and her care giver about self-care, newborn care, hygiene, contraceptive use, postnatal danger signs and when to return to the health facility [2, 3]. Immediate postpartum care is recognized as being key in the prevention, diagnosis, treatment of complications, and improvement of the quality of care [4, 5]. This is because most maternal and newborn morbidity and mortality occurs within this time frame. However, facility based care during this time period has been found to be sub optimal despite its importance for women and newborns [1, 6, 7].

Guidelines for clinical practice have been the focus of the World Health Organization (WHO) and the Ministry of health (MOH), in an effort to improve the quality of care over the last two decades [8, 9]. New recommendations for postnatal care disseminated by WHO were adapted by MOH Uganda in 2016 and 2022 [1, 10, 11]. These clinical guidelines were partially disseminated to all the districts in Uganda either as hard copy books or online through the ministry’s online platform [12]. The clinical guidelines for facility based immediate postpartum care include the monitoring of both the mother and baby, health education, assessments at discharge and advice on when to return to the health facility [1, 2, 9, 13].

The benefits of clinical guidelines in improving the quality of patient care and patient outcomes have been documented in several countries [14–17]. However, having practice guidelines may not always translate into provision of high-quality postpartum care or care based on the latest evidence. Rather, they may sometimes result in health workers feeling overwhelmed when faced with impractical guidelines especially in low resource settings [8, 15, 18, 19]. This could be because according to the guideline development group for the WHO guidelines for postnatal care, most of the recommendations were developed based on routine care practices in developed counties rather than strong evidence from research [13].

Our recent study on the facility readiness to provide immediate postpartum care showed that the dissemination of the MOH guidelines and their adoption into facility policies was low. We also found that many facilities lacked hospital policies and protocols for the provision of immediate postpartum care [20]. Over half of the rural facilities assessed had no scores for the routine monitoring of mothers and their newborns according to the MOH guidelines. Secondly, a recent review of the change in women’s access to postpartum checks in the immediate postpartum period found that despite the increase in the number of facility deliveries in Uganda, there is still a gap in their access to the recommended routine immediate postpartum care [6]. Studies done in other countries have shown that midwives’ perceptions of the clinical guidelines determine the use and success in the implementation of new clinical guidelines [15, 21]. Implementation of evidence-based practice is crucial for improvements in immediate postpartum care.

Nurses and midwives play a vital role in ensuring that patient care is provided according to the available evidence and the patient care protocols. The guidelines for immediate postnatal care were intended to be a guide to postpartum care provision for mothers and their newborns, hence establish the minimum standard of care for all clients. To ensure guidelines are implemented, an increased level of staff awareness of the guidelines, availability of trained staff, mentorship, and support from supervisors needs to be in place. Also, hindrances like the considerations of the cost and the motivation of the intended users of the guidelines need to be overcome [22–25]. Since the publication of the current Ugandan guidelines, there has been minimal research on how these guidelines are being used and what the midwives’ perceptions are about their use in the Ugandan context [26]. This study, therefore, sought to explore the midwives’ perceptions towards the use of clinical guidelines for the provision of immediate postpartum care within the greater Mpigi region of Uganda.

## Methods

### Study area

The data were collected through face-to-face in-depth interviews with the midwives from the selected health facilities in the greater Mpigi region (comprising of Mpigi, Butambala and Gomba districts) in the central region of Uganda. The midwives were from two hospitals, three health center IVs, and 32 health center IIIs (more information is summarized in Table 1).

**Table 1:** Characteristics of the health facilities where the study participants worked

Variable	Total (N = 40) n (%)	HCIII (N = 35) n (%)	HCIV (N = 3) n (%)	Hospital (N = 2) n (%)
District				
Mpigi	18 (48.65)	15 (46.87)	2(66.7)	1(50.0)
Gomba	10(27.02)	9(28.13)	1(33.3)	0(0.0)
Butambala	9(24.32)	8(25.0)	0(0.0)	1(50.0)
Health facility type				
Government	25(67.6)	22(68.75)	2(66.7)	1(50.0)
PNFP ^a^	7(18.9)	6(18.75)	0(0.0)	1(50.0)
PFP ^b^	5(13.5)	4(12.50)	1(33.3)	0(0.0)
Number of Midwives				
1 to 2 midwives	17(45.95)	17(53.1)	-	-
3 to 4 midwives	11(29.73)	11(34.4)	-	-
5 to 14 midwives	9(24.32)	4(12.5)	3(100.0)	2(100.0)
Number of deliveries per month				
1 to 20 deliveries	11 (37.5)	10(31.3)	1(33.3)	-
21 to 100 deliveries	22(52.5)	21(65.6)	1(33.3)	-
More than 100 deliveries	4(10.0)	1(3.1)	1(33.3)	2(100.0)
Number of postnatal beds				
No beds available	5(13.5)	5(15.6)	-	-
1 to 4 beds	22(59.4)	21(66.5)	1(33.3)	-
5 to 9 beds	7(18.9)	6(18.8)	1(33.3)	-
10 or more beds	3(8.1)	-	1(33.3)	2(100.0)
Participated in RBF ^c^				
Yes	32 (86.5)	28 (87.5)	2 (66.7)	2 (100)
No	5 (13.5)	4 (12.5)	1 (33.3)	0(0.0)

a- Private not for profit health facility

b- Private for Profit facility

c- Result based financing

### Study design

This was an exploratory descriptive qualitative study done to explore the midwives’ perceptions towards postpartum care. This approach was chosen because it is useful when a researcher desires to explore and describe participants’ perceptions especially in areas where little is known. It is also used in health care settings to answer health care questions specific to a given discipline [27, 28].

### Study participants

The study participants were midwives working in the postpartum units of the selected health centers and Hospitals in the three selected districts in the greater Mpigi region.

### Sample size and sampling method

We enrolled midwives employed in the greater Mpigi region who had been working at a health facility that conducted deliveries (Health center III, IV and Hospital) and provided postpartum care for at least three months at the time of the study. Written informed consent was sought from all midwives who were willing to participate in the study. All midwives who were on leave during the study period were excluded from this study. Study participants were purposively chosen to ensure representation of the different facility types and levels in the study. The midwives interviewed included facility in-charges, regional trainers and those providing bedside care, and this was done to ensure variation in the experience and characteristics of the participants. Participants were recruited until data saturation (when no new data was generated from the interviews) was reached. This was reached after 50 interviews; prior studies have suggested that 40–60 interviews would be a substantive sample size in qualitative studies [29–31].

### Data collection tool

Data were collected using face-to-face interviews with the midwives using an indepth interview guide. The interview guide was prepared by MN. GKN, DKK and KS after reviewing of the available literature. The guide topics included: social demographic characteristics, knowlegde of the postpartum care guidelines and perceptions about the postpartum care guidelines (importance, feasibility and barriers to their implementation).

### Data collection procedure

Data were collected by MN and three research assistants. Two of the research assistants were graduate nurse-midwives who had just completed their internship training, and one had a diploma in nursing with two years’ experience working as a midwife and several years’ experience in conducting interviews. All research assistants were trained for three days by MN in interviewing, communication skills, note taking and ethical conduct of research before the commencement of the data collection.

For facilities where more than one midwife was found at the station, respondents were purposively selected to have midwives with varied working experience to vary the respondents in the study. This was done because midwives with variations in working experience and age or location may have differing perspectives about postpartum care. All midwives who consented were interviewed at a convenient time chosen by them that wouldn’t interfere with the delivery of services to their clients. All interviews were conducted from a private place where there were minimal interruptions during the interviews. All participants were informed that the interviews would be audio recorded and their permission sought to record the interviews. Each interview took between 45 and 90 min to complete.

## Data management and analysis

MN listened to the audio interviews as soon as possible after they were recorded. The audio-taped interviews were transcribed verbatim and the transcripts checked with the written notes for consistency by MN, and the three research assistants. The transcripts were filed according to facility name, date and midwife’s number.

The transcripts were uploaded into NVivo software [32] by MN for ease of data management. Thematic analysis was done following the six steps of thematic analysis as proposed by Clarke and Braun [33]. MN read and re read the transcripts together with DKK and GKN to identify and highlight significant statements and quotes that provided understanding of the respondents’ perceptions about providing postpartum care using the guidelines. These significant statements were used to make clusters of meaning which helped MN, DKK and GKN to identify themes from the data. Agreement on the meanings of the data and emerging themes was ensured. The themes identified were further clarified and refined until consensus was reached. Some of the reported statements by study participants were used as quotes in the results section.

## Results

We interviewed 50 midwives, 14 were from hospitals, 3 were from health center IVs while 33 were from health center IIIs. Their average age was 28.7years (23–49 years), and the average working experience was 5.1 years. Majority 42(84%) of the participants were certificate holders while 8 (16%) had a diploma in midwifery. Only 1/8 (11.9%) of the diploma holders and 5/42 (12.5%) of the enrolled midwives knew about the guidelines and could explain them correctly. Midwives working in facilities that were participating in the result based financing (RBF) program stated that they had been reoriented to the postnatal guidelines through the program. More information regarding the characteristics of the health facilities where they worked are presented in Table 1.

**Fig. 1:**
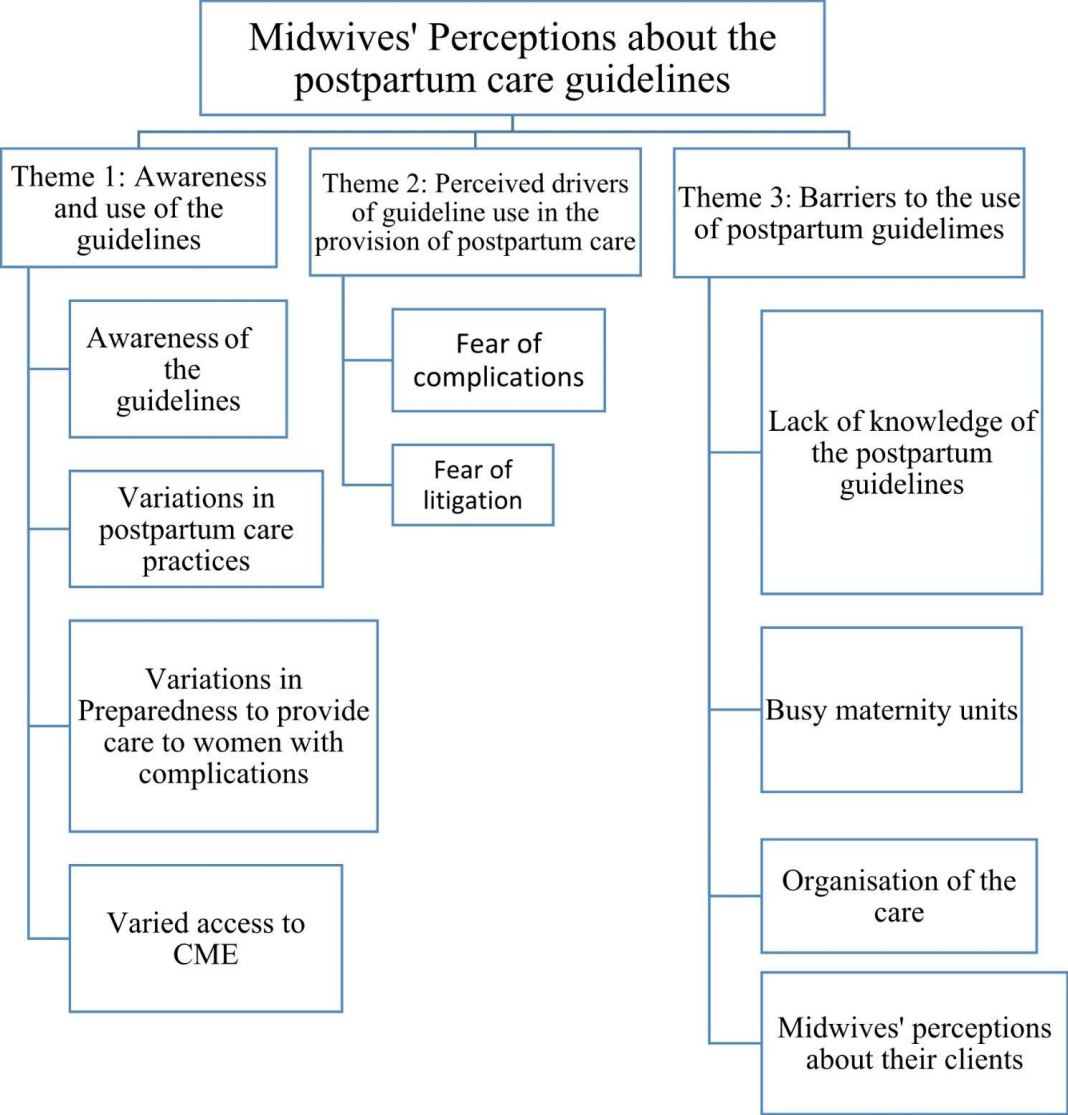
Thematic diagram of the midwives’ perspectives about the postpartum care guidelines

There were three themes that emerged from the data analysis namely; awareness and use of the MOH guidelines, and perceived drivers of guideline use and perceived barriers to using the guidelines (see Fig. 1). The subthemes under each theme are expounded in the paragraphs below.

### Theme 1: Awareness about and use of the new guidelines for postpartum care

There were four sub themes elicited under this theme namely; awareness of the guidelines, variations in the midwives’ practices, variations in the level of preparedness to care for women with complications, and varied access to continuing midwifery education. The midwives’ perceptions under these subthemes are broadened in the paragraphs that follow.

#### Subtheme I: Awareness of the guidelines

Often, there was a copy of the Uganda clinical guidelines (2016) [3] in the outpatient department (these guidelines are similar to the current guidelines which have been recently released in 2022). Some midwives even had the soft copy version on their phones for ease of access. However, some had not read the guidelines or were unsure of where the books were at the time of the interview. Other midwives had not yet received the updated guidelines four years after they had been written and disseminated and were using the older guidelines from 2012.

*“We might have the UCG but not here, this side of the facility. We (might) have our own copy here. But right now, this is what we have [“2012 guidelines]”, an older version of the Uganda clinical guidelines}”.* Midwife 35 health center III, Mpigi

*“We don’t have the new guidelines for postnatal (care). We are still following the routine care (monitor the woman and baby in the first hour then transfer them to the postnatal ward where the caretakers are instructed to report any complication. The mother baby pair may then be seen at the time of discharge if no complication arises during their hospital stay”* Midwife 13 hospital

We also found that some midwives had the latest guidelines at hand both as hard copies and as soft copy documents on their phones but were not able to articulate the key points in them.

*“I told you before that we have the UCG guidelines or are you thinking that they should be separated? [ have postpartum different from the general guide] We have them. Each person has them on their phone. No one is lacking them. You are telling me to go back and read them such that I know the key things”* Midwife 5 Health center IV

One of the reasons noted by the midwives for not being aware of the guideline contents was the fact that they were busy, lacked knowledge about the guidelines and most did not like reading as explained in the excerpt below;

*“They are there. We put them on the walls. We have a clinical guidelines book but it is not used. You know midwives are not friendly to reading. We have (a) copy in the labor suite. When a midwife crams something, it is not easy to change those people”.* Midwife, 8 hospital

#### Subtheme II: Variations in postpartum care practices

There were some variations in the way that the midwives perceived their ability to provide care to their clients in the immediate postpartum period. This was in light of the patient health education, monitoring, length of hospital-stay, the patient discharge criteria, and the midwives’ perceived preparedness to care for women with complications within the immediate postpartum period. These sub themes are explained further below.

#### Provision of health education

Health education of the mothers is part of ideal postpartum care, as it can provide information on postpartum expectations, nutrition, hygiene, perineal care, danger signs of postpartum and newborn complications, breastfeeding, and newborn care, among others. Some of the healthcare providers were aware of the need to provide health education and hence midwives with less busy units were able to prioritize this component of the care as exemplified by 2 respondents:

*“I emphasize the point of health education that I talked about, then I advise them to come back because I don’t tell them to go for forever and not return in hospital, we give them a return date”* Midwife 19 health center III

*“They need a demonstration of how a baby should be held when breast feeding.*” Midwife 13 Hospital

However, midwives within busy health units were unable to provide such education talks. Yet such education talks could provide advice on mother/baby nutrition, hygiene, baby care, and danger signs, among others

*“We are always busy thus failing to give them these health talks … we only discharge them, and they go home.”* Midwife 8 Hospital

#### Postpartum monitoring

The midwives were aware that the women needed to be monitored after delivery. Although their recall of the frequency of monitoring was varied, all agreed that the women and babies needed to be seen again after delivery. The interval for monitoring at some facilities were based on the previous now outdated UCG (2016)[34], which stipulated that monitoring be done quarter hourly in the first two hours after delivery whereas some practiced monitoring the women half hourly in the first two hours after delivery as stipulated in the new guidelines[10, 12, 35].

On inquiry about what care midwives provide and what guidelines they follow in providing postpartum care, the respondents emphasized the importance of timely client monitoring according to set guidelines, as noted by the two respondents below:

*“But all we do is we assess the mother, is the mother, okay? ‘Mother, how are you feeling?’ She might tell you that she has a terrible headache. Then there is an action there.” Midwife 5 Health center IV*

*“But now we monitor every 30 (thirty) minutes for example a mother has delivered at 11:00 am, we monitor at 11:30 then 12:00Midday we keep writing as we keep monitoring. For the mother we monitor bleeding, BP, temperature and then the pulse; for pulse check is very important. For the Baby we monitor…… breast feeding has the child breastfed”.* Midwife 15 health center III

However, respondents acknowledged that they often do not provide comprehensive postnatal care as indicated in the guidelines. Depending on how busy midwives are, the space available and how long postpartum mothers stay, midwives may not provide the ideal care. In the quotes below, a midwife explains how part of the monitoring role is shifted to the client and her attendants.

*Even when you get busy, you find time to check on them. Ask about the bleeding; how the baby is doing; breast feeding, baby’s cord. You encourage her to monitor the cord. Have you passed urine? What does the pad look like? Even if you did not really go there, you may ask while passing by. You send a few instructions to the attendants…* Midwife 36 Health center III

“… *you tell the mother that every woman who delivers has to lose blood however this blood should be minimal … if you are bleeding profusely, ask your attendant or immediate neighbor to alert us*” Midwife 9 Hospital

*“You advise the mother to breastfeed the baby immediately in the first 30 minutes. You also tell her to keep monitoring the cord for bleeding. She is told to alert us in case she sees any bleeding such that we can tighten it. We also offer her warm tea after delivery. After taking the tea, she can rest. Though she also is advised to keep passing urine”* Midwife 6 Health center III

#### Variation in duration of facility stay after delivery

The respondents noted that the duration of postpartum stay varied according to circumstances, even when guidelines for admission after delivery recommend that women and their babies be observed for at least 24 h. Facility stay in this study was between 4 and 48 h. This time was mostly driven by the number of patients versus the available space and beds. When the beds are few, the women and their babies are discharged quickly to make room for the newly admitted women or those who have just delivered. In other cases, the women requested to be discharged early due to overcrowding at the facility.

It’s their mentality after delivery, they think ‘now I am okay, I do not have any problem *‘now they think of their digging … their businesses…their cooking” Midwife 19 health center III*

*“Especially those who live near the facility, ‘she says I come from nearby, let me go home. I can return in case of anything wrong……they prefer to go home rather than sleep on the floor….” Midwife 11 Hospital*

The client’s experience also played a role in their requests for early discharge for example, the midwives noted that there were times when the clients complained about the long hospital stay because, ‘they did not have to stay long after their previous delivery.’ This highlighted the lack of uniformity in the length of hospital stay within the region.

*“They (the patients) complain, and they do not like it…they tell us when they deliver from other places, they go home immediately after delivery”* Midwife 2 health center III

#### Variation in patient discharge criteria

Concerning the criteria for discharging the mother baby pairs, midwives reported that they used their personal judgement and sometimes the mother’s condition or request to be discharged. This finding shows that care is based on the individual characteristics and needs of the client which is part of the recommendations for improving the quality of care. Women and babies were sometimes examined by the midwife before discharge, although this was not the practice everywhere.

*“No, we do not examine them. We only examine if a mother has a complaint at the time of discharge where she says she is not feeling well”.* Midwife 16 Health center III

*“This mother feels energetic. I take note of the rate at which she is bleeding. I cannot discharge her if she is still bleeding even if she requested with pressure. Bleeding is important. I also take her blood pressure. In most cases, after delivering, most mothers’ blood pressure increases. So, it is a good practice to monitor her blood pressure. Then for the baby, I check the cord. What is the cord like? Is the baby breastfeeding well? If not, I won’t discharge them. Before discharge, I examine her. It is a good practice. Sometimes she might be going yet she is bleeding”*. Midwife 1 health center III

There were cases where the midwife would have to discharge a woman before 24 h have elapsed and this was noted to be because of the pressures of being the provider and caretaker at home and the women’s sense of wellbeing, once they deliver their babies.

“…*some of the mothers have already produced … she tells you “musawo [Midwife], this is the amount of bleeding I usually have after delivering.” Even if you see the bleeding is still much, she just insists that the bleeding is okay for her … there is no cause for alarm, me I am going home.”* Midwife 5 health center IV.

#### Subtheme III: Varied preparedness to manage women with complications

Concerning the midwives’ perceived ability to provide care to women who develop complications during the postpartum period, the presence of equipment and supplies, ability to refer women and access to continuing midwifery education on emergency obstetric care was considered key by the midwives. A deeper discourse of these subthemes follows below;

#### Equipment and supplies

When asked about their ability to identify and manage some obstetric complications, the midwives said they were able to recognize these but how they managed the conditions depended on the equipment, drugs, and the number of staff available. This sometimes prompted them to refer the clients to other facilities, yet they could have attended to them, as noted by 3 respondents:

*“In postpartum care most times, blood pressure is important as well as the rate of bleeding, and blood pressure. Then for the baby, the cord. How prepared am I? It depends on what I have. When I have the items I need like misoprostol, hydralazine, and magnesium sulphate and the midwives are present I am prepared. You can’t manage an emergency alone musawo [midwife].”* Midwife 4 Health center III

*“Yes. for the mother, we take blood pressure and temperature. But now the BP machine’s cuff got spoilt. But we have been assessing them.” Midwife 4 health center III*

*“Supplies are not enough … for instance we do not have gloves and it is weird to ask a mother to buy gloves in order to get treatment”* Midwife 9 Hospital

*“I often find myself in a dilemma… the unit is new, and we have no equipment to resuscitate the babies… I often have to refer the women to the hospital and sometimes they think that I am lazy, and I just hate working….(eyes tearing)...”* Midwife 27 Health center III

Concerning the use of Chlorhexidine gel for umbilical cord care, midwives noted that they often do not have it so cannot teach the mothers cord care based on the new guidelines. One midwife elaborates on the issue below;

*“Yes, she can use (chlorhexidine gel) but some of these things are in the private set up. Chlorhexidine gel can be used but here in a government setting…. we might have some but you cannot give everyone. Just know that they will not be enough yet each mother requires her own. After using it on her baby…… you have to give it to her. Do you understand? So if you teach her the local technique, it is much better”* Midwife 10 hospital

#### Referral

The respondents recognized the need to refer women who were either suspected of being prone to getting complications or had developed complications to high level facilities that could provide comprehensive health care to the women.

*“If there is a cause of bleeding I call the ambulance for her to be transferred as she can bleed up to death” Midwife 36 Health center III*

*“If a woman has any condition requiring advanced care in this or her past pregnancies, I immediately refer her to the hospital. I do not want to have issues here….” Midwife 31 health center III*

The midwives at hospital recognized that they received many patients referred from lower health centers. Concurrently, there are several instances of self-referral among their clients that were observed especially among those patients that are referred to the national referral hospital.

*“This being a hospital, day shift is very busy because it covers many health centers … many people find it easy to come here even if they have been referred to Mulago Hospital” Midwife 9 Hospital*

#### Subtheme IV: Varied access to continuing midwifery education

When asked about continuing medical education, almost all the facilities said they have weekly and monthly sessions. However, many midwives said they had not had sessions on emergency maternal and newborn care or the new guidelines since they qualified.

*“I have not had a chance to go for any trainings since I qualified,,,, may be the In-charge and others have gone but for us we do not go…. Maybe they should organize some training to teach us about the new guidelines.”* Midwife heath center III

*“We have never received any and we are in dire need of those trainings. Personally, I have never been trained … we only get in-house CMEs but they are not on emergencies which we actually need”* Midwife 9 hospital

### Theme 2: Perceived drivers of guideline use in the provision of postpartum care

Under this theme the midwives expressed their perceived motivators to the use of the immediate postpartum care guidelines.

#### Subtheme I: fear of complications

One of the reasons noted for monitoring women was the fear that they could develop complications if not seen regularly. One midwife noted that even women without preexisting issues could turn for the worse as expressed in the quote below.

“*Yes. Still after delivery, you must assess your mother. Why? Because you may think that this mother is okay, and you leave her around there (PNC ward) and you come this way (labor ward). You see the distance from there up to here is a big one.” But all we do is we assess the mother. Is the mother okay?* Midwife 24 health center III

*“Sometimes you recall some things and notice that these mothers actually need care. Because even if she has delivered well and is doing things as per the ideal of breastfeeding well, feeling well, this mother’s condition may change in a split second. Therefore, I learnt my lessons from then. I can’t use the excuse of a big workload.”* Midwife 36 Health center III

#### Subtheme II: Fear of litigation

For some midwives the fear of litigation when complications occurred was the motivation for using the guidelines in the provision of care. One claimed that she did not want to lose her practicing license hence endeavored to do thing right.

***‘****It would be beneficial, just in case you have done everything, but it all fails, you have what to show, ……… I think that would be helpful in case you get a problem, you know the issues of midwifery you wake up one morning and your certificate is taken and yet you’re without blame.’* Midwife 2 HC III

### Barriers to the use of the immediate postpartum care guidelines

#### Sub theme II: lack of knowledge of the postpartum guidelines

It was noted that having hard copies of the Uganda clinical guidelines did not always result in the midwives knowing what exactly the guidelines had to say because either the midwives were too busy to read them, or they simply preferred in service training and mentorship sessions to bring them up to speed.

*“All things and even the guidelines, it is easier implementing if someone is knowledgeable. It is very difficult when one is not knowledgeable. On job training is very good since we are not many midwives. When you come for work, and teach the midwife while she works, it helps”.* Midwife 19 Health center III

Some of the respondents suggested that there should be some in-service trainings for them to aid them in understanding the new guidelines since they may not get enough time to read them. Respondents also pointed out the fact that they had not been oriented to the new guidelines, yet they felt a practical orientation to how to implement the guidelines was in order as some midwives at busy units remarked;

*“Yes. Guidelines are good but it also helps if someone has more knowledge. She might not have time to read through the guidelines but if someone comes and equips them (with updates), it helps”.* Midwife 19 Health center III

#### Subtheme III: Busy maternity units

Midwives in some of the busy facilities expressed concerns about the staff: patient ratios at the facilities which made it hard for them to provide care according to the guidelines especially in the postpartum period. Below is a quote from a midwife from a busy facility;

*“No, we have to be practical because you are the one on duty, you may have women in labor… if you do not expect any woman to deliver on labor ward … no woman in 2nd stage … by the time you move from labor ward to theatre and back …you just have to give up and do what you can …we talked to our supervisors about what is happening I do not know why they are not responding to our cry”* Midwife 9 Hospital”

*“….all time will have been spent on labor ward and by the time you remember that you had been assigned to work on postnatal, time will have passed like how you found me … the baby was very sick having asphyxia … will you remember the ward you were assigned to work?” Midwife 9 Hospital”*.

*“Due to the fact that sometimes when you are few on duty, you may come and find labor ward is too busy that you may fail to go to the postnatal ward). Though if you see that there is a mother who is really ill, even if you are too busy you have to. You may have time to pop in there even if you are too busy. A time may come when you fail to go there. And this one is delivering then this one too after the other. Your colleague is in the theater and you are two. All women want their babies; they are looking at you. And you have to make sure that these babies have life.”* Midwife 10 hospital

These busy maternity units may result in midwives with attitudes that are not good because they are constantly tired. This hinders the provision of quality care to the clients. One midwife narrates her colleague’s experience;

*“I once had a friend who told me that happened to her patient, so I asked her ‘did you monitor her?... and she told me no. And then I asked her, ‘you haven’t monitored a woman who is freshly delivered?.. She told me, she got tired (Nakowa!). That attitude of you got tired! “Nakowa”…*Midwife 2 health center III

Health education of the mother is part of ideal postpartum care. While the healthcare providers were aware of the need to provide health education, their busy schedules would not allow them to provide such education talks. Yet such education talks could provide advice on mother/baby nutrition, hygiene, baby care, danger signs, among others. This suggests that busy healthcare provider schedules were an obstacle to providing adequate postpartum care. These are exemplified by the respondents’ quotes below:

“We make it (health education) for a group. We may not do all of them (the topics) at once. Today we may health educate on diet, tomorrow immunization. If she has been staying on the ward, she will be picking if she is interested. By the time she leaves, she is equipped with all. Where possible we can do one on one especially with HIV positive patients. For them you have to go the extra mile like; for them to give the baby medicine and for herself care.” Midwife 10 Hospital

#### Sub theme IV: Organization of care

The midwives’ identified different aspects of the organization of the care that did not favor the provision of care using the MOH guidelines. These aspects included the setup of the maternity units, the distribution of the staff on each shift, and the prioritization of deliveries.

### Unit setup

Some of the units were set up as one unit with the high risk antenatal, labor ward, neonatal and the postnatal sections. This arrangement of the ward was both good in that the patients were seen by the same midwives throughout their care but sometimes led to the patients not receiving care according to the guidelines. This was common when the unit was very busy, because the postnatal and neonatal units are situated far from the labor ward making it hard for midwives to frequently check on the women and babies within those sections.

*“…but also, our supervisors know that the person who works in labor ward is the same person to work in postnatal, and in neonates … that is the situation, and we have no option….”* Midwife 8 Hospital

The midwives noted that their numbers were not commensurate with the patient load and were based on the designated staffing norms for public health units stipulated in 1999 [36]. The population has doubled since then and facilities have experienced a rise in patient numbers over the years but the number of midwives that are designated to work there has not been changed to match the new patient population [36]. This, they recognized as a deterrent to good quality care provision because the available midwives are unable to adequately provide care to the high patient numbers.

*“it is the same for the evening shift, we are only two midwives on duty… however; if we are not busy, we try our best to give the care though it is very difficult to routinely give it. There is need for division of labor so that we give that quality care” Midwife 9 Hospital*

*“Actually, we have ever complained to our administrators, and they said the staffing is just enough. they said they can’t add to the number, may be because of money issues…. those things”.* Midwife 10 Hospital

#### Distribution of the staff in the different shifts

Within most units, the number of midwives allocated to the dayshift was more than those allocated to the evening and nightshifts. This unequal distribution was attributed to higher patient numbers and administrative roles during the dayshift:

*“It depends…there are more midwives on day duty compared to evening and night shifts because there is a lot of work during daytime this being a hospital, dayshift is very busy because it covers many health centers….”* Midwife 9 Hospital

However, this arrangement sometimes leads to constraints for the midwives who work during the.

evening and nightshifts. This is because there are often only two midwives on duty, who are.

responsible for providing care to the laboring women, the sick newborns, those who have had a.

cesarean section, and any newly admitted women.

*“But on night duty you are only 2 … one can be on labor ward and another on postnatal … on labor ward women deliver at night and in the end the staff on postnatal has to come and assist on the labor ward and ends up working there the whole night.”* Midwife 9 hospital

To improve the patient care, some midwives wanted labor ward to be split into two separate units each with their own administrators and midwives, so that each midwife can concentrate on caring for specific clients, i.e., the midwife on the labor ward does care for women in labor only while the ones in the postnatal unit care for those in the postnatal section only but not both.

*“We wanted labor ward to remain labor ward and postnatal to become a unit of its own such that postnatal gets an in charge with their staff and even labor ward. But it is impossible* “Midwife 10 Hospital

#### Prioritization of deliveries

Midwives in busy facilities noted that they were forced to prioritize women that were delivering over the provision of other care to their clients. This was common during the evening and night shifts and busy times of the day.

*“You and your colleague have five mothers due for theatre so you have to go and assist her….all time will have been spent on labor ward and by the time you remember that you had been assigned to work on postnatal, time will have passed”* Midwife 9 hospital

*“That is what is on ground … you will see it especially on special duties…busy … we are usually busy at night …actually it is the busiest duty … you can decide to neglect some of the cases, not because it is what you wish … that is where your capacity ends …you are attending to a woman in second stage do you think you can go to one who is crying due to pain?”* Midwife 9 Hospital

### Subtheme V: Midwives’ perceptions about their clients

Under this subtheme, the midwives noted that the postpartum women’s perceptions about postpartum care like the perceived cost of staying at the health facility, perceived lack of comfort at the facility, the patients’ understanding of the importance of postpartum care and their traditional practices in the postpartum period led to the patients’ unwillingness to stay at the health facilities for 24 h as stipulated by the guidelines. More is said about these perceptions in the paragraphs that follow.

#### Cost of staying at the health facility

Sometimes, the midwives found it hard to implement the 24-hour patient monitoring of postpartum clients because the clients and their attendants preferred to go home earlier than the stipulated time. There were several reasons identified for this including the patient’s comfort, the extensive costs in time, expenses, and the need to continue with their multiple roles at home. Attendants and the clients complained about the extensive costs (buying diapers, food, and drinks for the mother and attendant, plus transport to and from the hospital for those bringing them refreshments) they incurred when they had to stay at the facility for 24 h. This would mean that they either request discharge or are unhappy when discharged later than they anticipated, according to the recommendation of the guidelines on maternal and newborn health, as shown by one respondent:

*“They don’t take it well at all (when asked to stay in hospital overnight), they say they use a lot of money to get things to eat, the place is remote, there are no edibles, we tell them that’s how we do it, they should fend for themselves.”* Midwife 2 health center III

#### Perceived lack of comfort while at the facility

Some midwives noted that the postpartum women preferred to go home soon after delivery because they felt more comfortable there since there was often overcrowding within the health facilities. Additionally, sometimes the amenities like bathrooms and toilets lacked running water making it inconvenient for the clients to use them. Yet early discharge is against the guideline recommendations. This is exemplified by one respondent’s observation;

*“The women, especially those from near the hospital prefer to go home where they are comfortable instead of sleeping on the floor after delivery. They often suggest that they be allowed to go home* [*before 24 hours have elapsed*] *and will return in case of any complication”.* Midwife 11 Hospital

#### Patients’ understanding of the importance of postpartum care

Midwives noted that women were often scared of the labor pains and rushed to the health facilities for care but as soon as they delivered the babies, they became agitated and desired to go home. Some midwives attributed this to a lack of understanding of the importance of facility-based postpartum care.

*“She comes in pain and desiring that you care for her but as soon as the baby is out,….while she is still on the delivery bed, she starts asking you when she can go home…. They do not understand why they need to stay here for a while.”* Midwife 38 hospital

*Even if you explain to her that she should stay, she insists on leaving and if you do not discharge her, she just leaves”* Midwife 26 health center IV

#### Traditional practices around the postpartum period

Some midwives recognized that the women’s desire for an early discharge was based on the need to get home and perform the culturally approved care for both the mother and baby (bathing the baby in herbs and giving the mother steam baths) as soon as possible. They perceived that the hospital environment was not conducive for this since they could not prepare or get the herbs needed for this while still at the hospital. So they request for early discharge from hospital to facilitate them to conduct the rituals in a timely way. This makes it difficult for the midwives to comply with the postpartum guidelines for routine monitoring of women and length of hospital stay

*“The women desire to go home so they can make kyogero* [*a herbal mixture used to bathe the baby in the first few months after delivery*] *and bathe their newborn and also for the mother to get her steam massage as is recommended culturally. That cannot be done while they are still here yet it is deemed to be urgent and very important…….* Midwife11 Hospital

## Discussion

This study explored the midwives’ perceptions on the provision of postpartum care. Although the national guidelines from the Ugandan Ministry of Health had been disseminated in 2016 and again in 2020, our study found that not all the facilities had or were using copies of the guidelines (hard and soft copies). In some facilities the guidelines were available but not routinely used due to healthcare providers’, client and facility factors as barriers [12].

The findings are similar to those from other studies: While considerable efforts are directed at developing and disseminating international guidelines to improve clinical management of maternal and newborn health in low-income settings, guidelines appear to rarely influence practice, and healthcare providers often do not provide evidence-based care. For instance, a facility based audit on facility based immediate postnatal care at Jinja regional referral hospital in Uganda found that the care was suboptimal and the practice guidelines were not routinely used in care provision [17]. Other studies around child birth practices also found routine implementation of the practice guidelines wanting [37, 38]. Furthermore, a systematic review on guideline use in low income countries found that knowledge and dissemination of the guidelines did not translate into their use during management of patients [39–41]. Other Ugandan studies have noted that, patients are not monitored, examined or health educated as stipulated when guidelines are inconsistently implemented [42, 43]. The main reasons for the failure to adhere to recommended guidelines are related to incomplete training coverage; inadequacies in setting up local standards, champions and leadership. Failure to recognize and appreciate good work or good performance; poor communication and lack of team teamwork; organizational constraints (such as organization of care, duty coverage and staffing norms) and limited resources (both financial and non-financial) are also notable barriers. Counterproductive health worker norms (such as absenteeism); absence of perceived benefits or incentives linked to adoption of new practices; difficulties accepting change and failure to adopt new changes; lack of client motivation; and conflicting attitudes and beliefs (of both the clients and healthcare providers) do hinder guideline adoption and were noted in this study [41, 44–46]. These barriers may largely be because guidelines are adopted from high income settings and there may be little or no involvement of the end user in the development of those that are developed in low income settings [47]. This may result in poor adaptation of the guidelines and their acceptance by those that are required to implement them. There has been a call for contextual research on postpartum care that can address the country and region specific guideline use gaps [1, 13].

Midwives in our study could connect the lack of guidelines and organizational barriers to the lack of facility readiness for management of obstetric emergencies and postpartum care. For example, they noted that it was sometimes not feasible to implement the guidelines and that some organizational changes would be required to enable their implementation (like the separation of the theatre and postnatal unit staff from the labor ward to ensure that duties were effectively covered). This has also been highlighted by J Nabyonga Orem, J Bataringaya Wavamunno, SK Bakeera and B Criel [48] who found that sometimes organizational changes need to be implemented to enable the smooth implementation of the guidelines. Similarly, the social set up and structure of the maternity units have been cited as hindrances to maternity staff care provision in Malawi [49].

Lack of time to search for information and the inability to make time during working hours to look for new information was noted as a barrier to utilization of evidence and new guidelines in our study. Although many midwives had smart phones and could have gotten or even had the clinical guidelines from the MOH website, many of them claimed that they had no time to read and some even felt those guidelines were only for the nurses. This could have come about because previous guidelines had been silent on many midwifery aspects unlike the current guidelines[12, 34, 35, 50]. Studies have found that lack of time, age and lack of interest also hindered the use of technology by health workers [19, 51]. Other reasons why the knowledge of the guidelines could have been limited involved the scarcity of guideline copies, the personalization of the available copies and the preferred format of the guidelines by the health workers [18].

Healthcare providers were not implementing evidence-based practice (EBP). The latter refers to the use of best, valid, currently available and relevant research findings, expert opinion, standard guidelines and books in clinical decision-making practice [52]. The provision of evidence-based health services ensures improved patient safety and the quality of care. When asked about their routine care practices the midwives highlighted that, the frequency of observation of the woman was based on how she presented (or circumstances) and not on the stipulated guidelines for care. While respondents perceived that implementation of practice guidelines is necessary to improve care, they often were practicing without considering them. Consequently, there were variations in the care provided, which has implications for quality of care experienced by the postpartum women. Besides, even where guidelines were available, protocols and job aides to simplify the guidelines were either not available or not routinely used [20]. Given that the WHO guideline development group recognized that many of their recommendations were based on routine practice in developed countries and low level evidence, contextual studies need to be done to establish the best practices for immediate postpartum care provision [1, 2]. This evidence should then be availed readily to nurses/ midwives in a form that aids them to recognize its implications to practice[19]. Also midwives should be involved in the process of protocol development in order for them to have a positive attitude towards the guidelines and their implementation[21].

The midwives believed that the women did not recognize the value of hospital based postpartum care but rather preferred to be at home with their families which they honored. This was in line with what other authors have found that midwives tend to base their decisions on a mix of the clinical evidence, their expertise and the values of the woman[21]. The variations in the postnatal care provided is in agreement with a review to assess the use of postnatal health-care services in low and middle-income countries, where use of postnatal care services was found to be highly inequitable and varied markedly with socioeconomic status, between facilities in the same country and region, and between urban and rural residents [45]. This may be related to absence of management protocols, failure to use available guidelines, and variation in facility readiness (absence of necessary medications, equipment, infrastructure, staff numbers, staff skills and poor staff motivation) [7, 20].

The results suggest that personal, interpersonal and institution barriers exist regarding adoption or implementation of recommended WHO guidelines on maternal and newborn health. This finding concurred with reviews which found that while the barriers identified are broadly similar in theme to those reported from high-income settings, their specific nature often differs as it is contextual [44, 47]. For instance, at an individual level, lack of interest in the evidence supporting guidelines, poor motivation for using the guidelines, subjective feelings that following new recommendations may erode interpersonal relationships with clients or erode professionalism, and expectations of providing individualized care may threaten successful implementation [44]. In contrast, at the institutional level, there may be lack of systems to introduce, adopt, adapt or reinforce guidelines [41]. There may also be poor teamwork across different cadres of health worker, and failure to confront or address poor practice [41, 53]. Adopting new guidelines requires focus on improving the motivation, relationships, working culture and environment [41, 49].

The prioritization of labour over postpartum care by the midwives in this study exposes the lack of continuity of care, further exemplified by midwives in our study who stated that women were discharged early because they requested to go home early. Although in some settings it was due to the high patient nurse ratios, the practice was similar even in facilities with few patients. This could be due to the non-recognition of the importance of care provision during this time in the prevention of both maternal and newborn morbidity and mortality [54]. Midwives also tend to pay more attention to post cesarean-section women compared to those who have had a vaginal delivery [54]. Postnatal care (PNC) ideally should be provided within 48 h to all women delivering in a health facility and their newborns [1, 10]. Providing continuity of skilled care throughout pregnancy, labor and delivery, and the early postnatal period has potential to reduce the risk of death or disability for both the mother and baby [55]. Multiple studies from Africa and Asia have found that delivering in a facility and/or being attended by a skilled provider during childbirth increases the likelihood that women and babies will receive early PNC [56–59]. However, this can only happen when the facilities, midwives and the clients recognize its importance.

In summary, there was limited support for the implementation of evidence-based practice by midwives. The lack of knowledge and skill to use the guidelines was related to busy schedules, high patient numbers, patient expectations to leave health facilities on request, lack of motivation, the lack of resources and perceived lack of continued medical education. The study also shows the missed opportunities to provide adequate immediate postpartum care for a positive experience. Possible postnatal interventions for the mother include; (i) iron and folic acid supplementation for at least three months; (ii) screening for and treatment of infection, hemorrhage, thromboembolism, postnatal depression and other conditions; (iii) prophylactic antibiotics given to women who have third- or fourth-degree perineal tears; and (iv) counselling on early and exclusive breastfeeding, nutrition, birth spacing and family planning options including any available contraception [45]. In a study to assess the provision of evidence-based preventive and health promotion targeted interventions to women, and subsequently their newborns, during childbirth in a high-mortality setting, most women did not receive the recommended routine processes of childbirth care. They and their newborns needed to benefit from their choice to deliver in a health facility, and even fewer benefited from even basic risk assessments, leading to missed opportunities to identify risks [60, 61].

Trustworthiness in qualitative research has been said to consist of four criteria, including; credibility, transferability, dependability, and confirmability [62]. To ensure credibility in this study, a clear description of the research context, problem identification, clear problem, appropriate data collection procedures, appropriate participant selection, acceptable data analysis and correct interpretation of the data. Transferability was established by providing readers with evidence that the research study’s findings could be applicable to other similar settings, contexts, situations, times, and populations. Dependability was achieved by having clearly described research methods, including participant selection, data collection, analysis and interpretation and providing adequate contextual information about the study setting. This can ensure that the study could theoretically be replicated by other researchers and generate consistent results. Confirmability was assured during the transparently described data analytical process, with a clear trail showing how data were collected, checked, and rechecked throughout data collection and analysis to ensure that emerging similarities and patterns were identified, and that results obtained would likely be repeatable by others who conduct research in a similar approach in a similar context. During the intervening time, there was concurrent data collection for another study on the processes involved in providing care within the postpartum period at three of the facilities which provided an avenue to ascertain whether what was reported by the midwives was true. The procedures followed in this study have been written clearly to ensure that they can be repeated in other contexts.

### Recommendations for patient care, research, and policy

First, midwives and other health workers should be trained in the use of guidelines whenever available. Proper planning and financing is key to ensure that quality of care can be maintained and enhanced. The focus should shift from just increasing the coverage of facility birth to improving the quality of care and to developing appropriate metrics to measure and track this progress. Facility readiness measures including the use of patient management guidelines may be one of the ways to improve the quality of care and the experience of care beyond the provision of care. Secondly, good quality care includes the timely and appropriate use of evidence-based clinical and non-clinical interventions that are acceptable to women. So skilled providers should not only be availed with harmonized patient management guidelines but should be motivated to use them in decision-making for patient care, through developing protocols and job aids. Global efforts to reduce preventable maternal and newborn mortality that focus on skilled attendance at birth should ensure availability of human resource, training, provision of harmonized guidelines, motivation to use the updated guidelines and improvement in facility readiness (provision of necessary supplies, medication, equipment, and infrastructure) for patient care. We should ensure that all health facilities fulfill the requirements and are capable of providing lifesaving emergency obstetric and newborn care.

The practice implication of our study is that the lack of universal use of patient management guidelines implies that the necessary postpartum care, including health education, observations, risk assessment and postpartum checks are not performed consistently. This combined with the short hospital stays after birth in sub-Saharan Africa, likely contributes to under-diagnosis and poor management of some birth complications [63–65]. Consideration should be given to effective coverage of postpartum care, according to the standard patient management guidelines including functions, content, and quality of care. The recent WHO guidelines recommend ANC, intrapartum, and postpartum services to facilitate a positive pregnancy experience. Recommendations for more structured and person-centered postnatal care contacts could complete the pregnancy cycle and improve both the woman’s experience and health outcomes. Notably, pre-discharge checks and health education are a key opportunity to advocate for greater use of services across the full 6-week postpartum period, and for emphasizing the danger signs of maternal and newborn illness (that are the major cause of early neonatal and maternal morbidity and mortality), and significantly provide or enhance continuity of care as well as the integration of maternal and newborn services.

### Strengths and limitations

We have used direct quotes to provide depth and enrich the data, and although the study was conducted in one region of the country, it provides insights that can be used as transferable knowledge to similar contexts in Uganda and other low-income countries. We had midwives from health center III, IV and hospitals, public and private facilities that gave us varied perspectives from different levels of care. We recommend that further studies be conducted in urban areas and that the perspectives of other health workers and stakeholders on postpartum care be explored.

## Conclusions

The provision of guidelines may not translate into improved provision of care. Global efforts to reduce preventable maternal and newborn mortality that focus on skilled attendance at birth should ensure availability of human resource, training, provision of guidelines, motivation to use the updated guidelines and improvement in facility readiness (provision of necessary supplies, medication, equipment, human resource and infrastructure) for patient care. Adopting and consistently using the new guidelines requires a focus on improving the personal motivation, nurturing positive midwife-patient relationships, and fostering a supportive working culture and environment.

## Data Availability

The data generated and used during the current study are not yet publicly available because the corresponding author is still using them for her PhD studies but can be availed by the corresponding author on reasonable request.

## List of abbreviations

MOH: Ministry of health
RBF: Result based financing
SARA: Service availability and readiness Assessment
WHO: World health organization
